# Supplementary material for: WeChat-assisted strategies for personalized health management in patients with AECOPD: A randomized controlled trial
Source: PLoS One. 2025 Aug 18;20(8):e0330673. doi: 10.1371/journal.pone.0330673 (PMC12360646; doi:10.1371/journal.pone.0330673)
Supplement: Attachment — Submitted filename: Comments.docx [file pone.0330673.s005.docx]

This is a highly meaningful study exploring the effectiveness of digital healthcare interventions utilizing WeChat in the context of the increasing AECOPD patient population in China and the growing importance of self-management. However, to enhance the methodological rigor and validity of the results interpretation, the following improvements are recommended:

1. A more detailed explanation of the Self-Management Ability Score is needed. Please provide the scoring weights and theoretical rationale for each sub-item (symptom management, daily life management, emotional management, information management, and self-efficacy). Considering that the WeChat intervention primarily focuses on communication with healthcare providers, symptom monitoring, and health information provision, the theoretical basis for including emotional management and information management as evaluation items is unclear. Additionally, please clarify whether "information management" specifically refers to health information management.
2. Please provide the theoretical rationale for using Kolcaba's GCQ to measure Quality of Life Score and its applicability validity for AECOPD patients. If you used an existing validated instrument, please add appropriate references and report the reliability coefficient obtained in this study.
3. Although baseline homogeneity was ensured through stratified randomization, simple t-tests and chi-square tests alone are insufficient for complete control of potential confounding variables. Please specify the variables corresponding to "etc." in "gender, age, disease duration, etc." (line 105, page 4) and consider additional control through multivariate analyses such as ANCOVA or multiple logistic regression.
4. The current study only performed between-group comparisons at the post-intervention time point. However, to evaluate the pure effect of the WeChat intervention, it is necessary to control for natural changes over time. Analysis comparing pre-post changes using difference-in-differences approaches or pre-post comparison methods is required to properly assess intervention effectiveness.
5. Please present the reliability (Cronbach's α, test-retest reliability) and validity (content validity, construct validity) verification results for the hospital-developed questionnaire used to measure patient satisfaction.
6. All major outcome variables except readmission rates and emergency department visits were measured through self-reporting. This increases the possibility of various response biases, including social desirability bias, response set bias, and expectancy effects related to the intervention. Particularly, the WeChat user group may be more likely to respond positively to new technology. Please describe measures implemented to minimize such biases (e.g., inclusion of reverse-scored items, response pattern analysis, correlation verification with objective indicators).
7. This study is a human subjects intervention research requiring strict ethical oversight. While approval from the Ethics Committee of the Second People’s Hospital of Fuyang City and adherence to the Helsinki Declaration are appropriate, a clear explanation regarding the equivalence of this approval to IRB (Institutional Review Board) or Independent Ethics Committee approval—as required by international journal publication standards—is necessary. Please provide supplementary information on whether the composition, independence, and review processes of the ethics committee comply with international standards (e.g., ICH-GCP guidelines).
