# Supplementary material for: WeChat-assisted strategies for personalized health management in patients with AECOPD: A randomized controlled trial
Source: PLoS One. 2025 Aug 18;20(8):e0330673. doi: 10.1371/journal.pone.0330673 (PMC12360646; doi:10.1371/journal.pone.0330673)
Supplement: Attachment — Submitted filename: Paper manuscript_with line number.doc [file pone.0330673.s006.doc]

**WeChat-Assisted Strategies for Personalized Health Management in Patients with AECOPD: A Randomized Controlled Trial**

LIli Zhou,Cailing Song,Wenpeng Xu,Ruirui Wang,Wei Zhang*

No.2 People's Hospital of Fuyang City,Fuyang Infectious Disease Clinical College of Anhui Medical University,Fuyang 236000,Anhui,People's Republic of China

*Correspondence:Wei Zhang,No.2 People's Hospital of Fuyang City,No.1088,West Yinghe Road,Fuyang,Anhui,People's Republic of China,Email [94499420@qq.com](mailto:94499420@qq.com)

**Abstract**

**Objective**

To investigate the application effect of personalized health management strategies based on the WeChat platform in improving the overall health status of patients with Acute Exacerbation of Chronic Obstructive Pulmonary Disease (AECOPD), aiming to provide scientific evidence for enhancing the health management of AECOPD patients.

**Methods**

From February 2024 to September 2024, a total of 120 AECOPD patients treated in the Comprehensive Ward of Respiratory and Critical Care Medicine at the Second People's Hospital of Fuyang City were selected. They were divided into an observation group and a control group, each consisting of 60 patients, using the random number table method. The control group received conventional care, while the observation group additionally utilized the WeChat platform for personalized health education, symptom monitoring guidance, patient-doctor interaction, and other interventions. Post-intervention comparisons were compared between the two groups regarding self-management ability scores, quality of life scores, readmission rates, and emergency visit frequencies.

**Results**

After the intervention, the self-management ability score of the observation group was significantly higher than that of the control group, with higher scores in symptom management, daily life management, emotional management, information management, and self-efficacy. Quality of life was also better in the observation group compared to the control group, particularly in physiological, psychological, sociocultural, and environmental aspects. The readmission rate was 8.33% (5/60) and emergency visits were 6.67% (4/60) in the observation group, both significantly lower than the control group's 21.67% (13/60) and 20.00% (12/60). Nursing satisfaction in the observation group reached 93.33%, significantly higher than 76.67% in the control group, with all differences being statistically significant (P<0.05).

**Conclusion**

The auxiliary management model based on the WeChat platform effectively enhances the self-management capabilities of AECOPD patients, improves their quality of life, reduces hospital readmissions and emergency visits due to disease exacerbations, increases nursing satisfaction, optimizes medical resource allocation, and promotes long-term health management among patients, thus possessing substantial clinical promotion value.

**Keywords** Acute Exacerbation of Chronic Obstructive Pulmonary Disease ; WeChat Platform; Nursing

**Introduction**

Chronic Obstructive Pulmonary Disease (COPD) is a common chronic condition characterized by persistent respiratory symptoms and airflow limitation [1,2]. Its acute exacerbation phase, known as Acute Exacerbation of Chronic Obstructive Pulmonary Disease (AECOPD), not only severely impacts patients' quality of life but also increases the burden on healthcare resources and socioeconomic costs [3]. In China, AECOPD is relatively prevalent, especially among individuals over 40 years old. Smoking, air pollution, and winter respiratory infections are primary triggers [4]. Due to colder climates and industrial pollution, the incidence rate in northern regions is higher than in southern areas. The frequency and severity of AECOPD episodes are closely related to patients' self-management abilities; effective self-management can significantly reduce the number of acute exacerbations, improve health conditions, and lower hospitalization rates [5].Current health management models faces numerous challenges, particularly in meeting personalized needs, long-term management, and real-time monitoring. Traditional nursing models often rely on face-to-face interactions or telephone follow-ups, making it difficult to continuously track and support patients' daily lives [6]. With the development of information technology, especially the popularization of mobile internet technology, social platforms like WeChat have been widely applied in health management due to their immediacy, interactivity, and convenience [7,8]. The WeChat platform can serve not only as a bridge for communication between doctors and patients but also facilitate the dissemination of personalized health education content and provide real-time guidance on symptom monitoring, helping patients better understand their conditions and master correct self-management skills [9]. Studies have shown that interventions based on the WeChat platform have positive effects on enhancing the self-management capabilities and quality of life of patients with chronic diseases [10-12].

Currently, there is limited research on leveraging the WeChat platform to enhance comprehensive health management for AECOPD patients. This study aims to investigate the application effects of personalized health management strategies based on the WeChat platform in improving the overall health status of AECOPD patients. This includes enhancing patients' self-management capabilities, optimizing symptom monitoring and feedback mechanisms, strengthening patient-doctor interactions, and improving quality of life. By comparing and analyzing the impact of traditional nursing models versus integrated management models incorporating the WeChat platform on patients' self-management abilities, this study evaluates the potential of this approach in improving health management for AECOPD patients. It lays the groundwork for further exploration of information technology applications in chronic disease management.

**Materials and methods**

**General information**

Based on previous studies[13,14], we assumed a 20% improvement in self-management ability scores with WeChat intervention. With α=0.05 and β=0.20 (power=80%), a minimum of 50 patients per group was required. Accounting for a 10% dropout rate, 60 patients were enrolled in each group.A total of 120 AECOPD patients treated in the Comprehensive Ward of Respiratory and Critical Care Medicine at the Second People's Hospital of Fuyang City between February 2024 and September 2024 were selected for this study (Figure 1). The study received comprehensive review and formal approval from the Ethics Committee of the Second People’s Hospital of Fuyang City (Approval Number: 20231112034) and strictly adhered to the relevant provisions of the Helsinki Declaration. All participants provided written informed consent. Patient data were handled according to ethical standards to ensure confidentiality and anonymity. No personal identifying information was used during analysis or reporting. For participants with limited literacy, the consent form was read aloud by a researcher, and a witness (independent healthcare staff) confirmed their voluntary agreement via signature. The ethics committee waived the need for parental/guardian consent as all participants were adults (aged 38–75 years).

**Figure 1** Flowchart

Inclusion criteria were: (1) meeting the diagnostic criteria for AECOPD as outlined in the "Chinese Guidelines for Diagnosis and Management of Chronic Obstructive Pulmonary Disease in Primary Care (2024 Edition)" [15]; (2) aged between 38 and 75 years; (3) possessing basic skills for using smartphones and WeChat applications; (4) voluntarily signing an informed consent form. Exclusion criteria included: (1) having other severe systemic diseases such as heart, liver, or kidney disorders; (2) suffering from mental disorders or cognitive impairments that affect self-management capabilities; (3) being unable to complete the entire research process.

Patients were randomly divided into observation and control groups of 60 each using a computer-generated random number table. To ensure comparability between the two groups, stratified randomization was employed, with patients stratified by gender and age, and then randomly assigned within each stratum to either the observation group or the control group. There were no statistically significant differences between the two groups in terms of gender, age, disease duration, etc. (P>0.05), ensuring comparability (Table 1).

**Table 1** Baseline characteristics comparison

| Characteristic | Observation Group （n=60） | Control Group（n=60） | Statistical value | *P* value |
| --- | --- | --- | --- | --- |
| Age(years,mean±SD) | 63.77±6.75 | 64.92±7.96 | -0.853 | 0.395 |
| Gender[n(%)] |  |  | 0.363 | 0.547 |
| Male | 44（73.33） | 41（68.33） |  |  |
| Female | 16（26.67） | 19（31.67） |  |  |
| Duration of Disease(years,mean±SD) | 5.12±2.32 | 4.95±2.11 | 0.420 | 0.675 |

**Nursing methods**

**Control group**

The control group received conventional nursing care, which included medication treatment, health education, discharge guidance, and other routine nursing activities.

**Observation group**

On top of the conventional nursing care, the observation group integrated comprehensive intervention management via the WeChat platform to ensure that each patient received a full six-month intervention period. Specific measures are as follows:

(1)Establishment and Functions of the WeChat Platform. ① A member from the medical team who is proficient in WeChat operations and possesses excellent communication skills was selected as the dedicated administrator for the WeChat platform. This individual underwent relevant training to manage and maintain the WeChat group on a daily basis, including responsibilities such as information dissemination, answering questions, and data recording. ② Using a departmental public mobile phone, a WeChat group named "AECOPD Health Management" was created. Members of this group include doctors, nurses, patients, their families, and research team members. Each patient and their family members were ensured to join the group, and they were guided on how to use WeChat for communication and data submission. ③ Before 9 AM every day, the administrator posts daily health tips in the group, such as medication reminders and breathing exercise instructions. Every Tuesday and Thursday, articles or videos about the basics of AECOPD, the latest research developments, and preventive measures are shared with the group. Patients can ask questions at any time within the group, and healthcare professionals respond within 24 hours, ensuring the accuracy and timeliness of the information provided.

(2) Personalized Health Education. ① Based on each patient's condition, age, lifestyle, and other factors, doctors and nurses jointly develop personalized health education plans. These plans are sent to patients via WeChat official accounts or mini-programs and are regularly updated and adjusted. ② An online health seminar is organized once a month, where experts are invited to discuss AECOPD prevention and treatment knowledge, with a Q&A session to encourage patient questions and participation in discussions. Seminar notifications are posted in the WeChat group one week in advance. After the seminar, patient feedback is collected to improve future content. ③ Patients are required to record their daily symptom changes (such as cough frequency, sputum color, degree of breathlessness), medication usage, and lifestyle adjustments (such as exercise levels, dietary habits, etc.). Patients must upload their health diaries to the WeChat group before 8 PM, where they are reviewed by responsible nurses who provide feedback.

(3) Symptom Monitoring and Feedback. ① Each patient is provided with an electronic symptom log, which includes key indicators such as cough frequency, sputum color, and degree of breathlessness. Patients are required to fill out this log daily and upload it to the WeChat group before 8 PM, allowing healthcare professionals to monitor symptoms in real-time. ② Healthcare professionals analyze the symptom data reported by patients. If any abnormalities (such as worsening symptoms) are detected, they immediately contact the patient via WeChat to provide targeted advice or adjust the treatment plan, documenting the process accordingly. In cases where patients exhibit acute exacerbation symptoms, they can use a one-click emergency service feature on WeChat to call for immediate medical assistance and simultaneously notify the attending physician to ensure timely treatment.

(4) Patient-Doctor Interaction. ① Each patient is assigned a responsible nurse who manages daily communication and care. Patients can consult their responsible nurse about their condition via WeChat at any time to receive personalized health guidance. ② A telephone or video follow-up is scheduled every two weeks to monitor the patient's recovery progress, answer questions, and adjust health plans as necessary. After each follow-up, the patient’s feedback is recorded, and continuous tracking of their health status is conducted. ③ A satisfaction survey is conducted monthly to gather patients' opinions and suggestions regarding the service content. Management models are continuously optimized based on survey results to improve service quality.

(5) Data Management and Privacy Protection. All data transmitted through the WeChat platform is encrypted to ensure patient privacy and security. Posting permissions within the WeChat group are restricted to healthcare professionals and administrators to prevent interference from irrelevant individuals. Chat records and health data submitted by patients in the WeChat group are regularly backed up for subsequent analysis and review.

**Observation indicators**

(1) Self-Management Ability Score: The COPD Self-Management Scale evaluates patients' self-management behaviors and capabilities. It encompasses symptom management, daily life management, emotional management, information management, and self-efficacy. Using the Likert scale method, scores range from 1 to 5, where 1 indicates complete disagreement or absence, and 5 indicates complete agreement or presence. The total score ranges from 51 to 255 points, with higher scores reflecting better outcomes.

(2) Quality of Life Score: The General Comfort Questionnaire (GCQ), developed by American comfort care expert Katharine Kolcaba, assesses individual comfort levels. It covers four domains: physiological (5 items), psychological health (10 items), sociocultural (8 items), and environmental (7 items), making a total of 30 items. Scores are rated on a four-point scale: 1 for "very unacceptable," 2 for "unacceptable," 3 for "acceptable," and 4 for "fully acceptable." Higher scores indicate better quality of life.

(3) Rehospitalization Rate and Emergency Department Visits: The rehospitalization rate and the frequency of emergency department visits due to acute exacerbations are statistically analyzed for both groups before and after the intervention.

(4) Patient Satisfaction: The Nursing Satisfaction Questionnaire, developed by our hospital, is used to assess patient satisfaction in both groups. The questionnaire has a total score of 100 points, categorized as follows: below 60 points as dissatisfied, 60 to 80 points as fairly average, 81 to 90 points as satisfied, and above 90 points as highly satisfied. The formula for calculating nursing satisfaction is: Nursing Satisfaction = (Number of Satisfied + Number of Highly Satisfied) / Total Number of Cases * 100%.

**Statistical analysis**

Statistical analysis was performed using SPSS 26.0 software. For continuous data, values are expressed as mean (x̄) ± standard deviation (SD), and the t-test was used to compare and evaluate the mean differences between the two groups. For categorical data, values are presented as [n(%)], and the chi-square (χ²) test was used for comparisons and analyses. A significance level of *P*<0.05 indicates a statistically significant difference.

**Results**

**Comparative analysis of self-management ability scores between the two groups**

After the intervention, the self-management ability score in the observation group was significantly higher than that in the control group. Scores for symptom management, daily life management, emotional management, information management, and self-efficacy were all higher in the observation group compared to the control group, with statistically significant differences (*P*<0.001) (Figure 2).

**Figure 2** Bar chart of self-management ability scores between the two groups

**Comparative analysis of quality of life scores between the two groups**

After the intervention, the quality of life scores in the observation group were higher than those in the control group. Specifically, scores for physiological, psychological, sociocultural, and environmental domains were all higher in the observation group compared to the control group (*P*<0.001) (Figure 3).

**Figure 3** Bar chart of quality of life scores between the two groups

**Comparative analysis of rehospitalization rates and emergency department visits between the two groups**

The rehospitalization rate for patients in the observation group was 8.33% (5/60), and the rate of emergency department visits was 6.67% (4/60). Both rates were significantly lower than those of the control group, which had a rehospitalization rate of 21.67% (13/60) and an emergency department visit rate of 20.00% (12/60). The differences were statistically significant (χ²=4.183, *P*=0.041 for rehospitalization; χ²=4.615, *P*=0.032 for emergency department visits) (Table 2).

**Table 2** Comparative analysis of rehospitalization rates and emergency department visits between the two groups

| Group | Rehospitalization rate | Emergency department visit rate |
| --- | --- | --- |
| Observation[n(%)] | 5 (8.33) | 4 (6.67) |
| Control[n(%)] | 13 (21.67) | 12 (20.00) |
| χ² value | 4.183 | 4.615 |
| P value | 0.041 | 0.032 |

Comparative analysis of nursing satisfaction between the two groups

**Comparative analysis of nursing satisfaction between the two groups**

The nursing satisfaction rate for patients in the observation group was 93.33% (56/60), which is significantly higher than the control group's rate of 76.67% (46/60). The difference was statistically significant (χ²=6.536, P=0.011) (Table 3).

**Table 3** Comparison of patient satisfaction between the two groups

| Group | Very Satisfied | Satisfied | Average | Dissatisfied | Overall Satisfaction |
| --- | --- | --- | --- | --- | --- |
| Observation (n=60)[n(%)] | 30 (50.00) | 26 (43.33) | 3 (5.00) | 1 (1.67) | 56 (93.33) |
| Control (n=60)[n(%)] | 20 (33.33) | 26 (43.33) | 10 (16.67) | 4 (6.67) | 46 (76.67) |
| χ² Value | - | - | - | - | 6.536 |
| P Value | - | - | - | - | 0.011 |

**Discussion**

AECOPD is a significant cause of high morbidity and mortality worldwide [16-18]. Effective self-management is crucial for reducing acute exacerbations, improving quality of life, and lowering healthcare costs. Traditional care models often fail to meet patients' personalized needs, particularly in terms of long-term management and real-time monitoring. In recent years, with the development of mobile internet technology, social platforms such as WeChat have gradually become new tools for health management due to their convenience and immediacy.

This study found that the self-management ability scores of patients in the observation group were significantly higher than those in the control group, as evidenced by improvements in symptom management, daily life management, emotional management, information management, and self-efficacy. This indicates that the WeChat platform not only provides personalized health education content but also enhances patient engagement and adherence through interactive communication [19]. Regularly pushed disease knowledge, medication guidance, and rehabilitation training videos within the WeChat group helped patients better understand their conditions and acquire proper self-management skills. The one-on-one doctor-patient communication channel allowed patients to consult doctors at any time, receive timely feedback and support, thereby enhancing their sense of trust and self-efficacy, which promoted the improvement of their self-management abilities.The quality of life scores of patients in the observation group were significantly better than those in the control group, further validating the positive role of the WeChat platform in improving patients' quality of life. GCQ scores showed significant improvements in physiological, psychological, sociocultural, and environmental aspects. Continuous health monitoring and timely interventions provided by the WeChat platform helped patients detect and manage changes in their condition early, reducing the suffering and inconvenience caused by acute exacerbations [20,21]. Online lectures and expert Q&A sessions offered abundant informational resources, helping patients build confidence in overcoming their diseases and enhancing their overall quality of life. Studies have shown [22] that in the long-term management of COPD patients, social media platforms like WeChat may be considered a sustainable strategy for implementing health-related quality of life improvements.

The rehospitalization rate and emergency department visit rate for patients in the observation group were 8.33% and 6.67%, respectively, both lower than the control group's rates of 20.0% and 18.3%. This indicates that the management model based on the WeChat platform effectively prevents acute exacerbations of AECOPD, reducing unnecessary hospitalizations and emergency visits. By recording symptoms daily and uploading them to the platform via WeChat, healthcare providers can adjust treatment plans according to the patient's actual condition, preventing disease progression. The health education content and symptom monitoring guidance provided on the WeChat platform enhance patients' self-monitoring abilities, enabling them to take timely and effective measures when mild symptoms occur, thereby reducing the risk of acute exacerbations [23].Patient satisfaction with the management model in the observation group was as high as 93.33%, significantly higher than the control group's 76.67%. This suggests that the WeChat platform not only improves the quality of medical services but also enhances patient satisfaction. The convenience and interactivity of the WeChat platform allow patients to access needed information and services anytime and anywhere. Close communication and timely feedback between doctors and patients increase patients' trust and sense of belonging, which not only improves adherence but also lays a solid foundation for future promotion of this model.

The depth and frequency of patient-doctor interaction in our study surpassed that of previous interventions. Patients could consult their assigned nurses at any time via WeChat, and biweekly telephone or video follow-ups were conducted to monitor progress and adjust health plans. This high level of interaction significantly enhanced patient trust and adherence, contributing to the observed improvements in self-management and quality of life. Our model emphasized long-term sustainability through monthly online health seminars and expert Q&A sessions, which provided patients with continuous access to updated knowledge and support. This long-term management approach, combined with the convenience and interactivity of the WeChat platform, represents a significant advancement over existing interventions and offers a scalable solution for chronic disease management.

**Limitations**

This study also has certain limitations. The sample size was relatively small, which may affect the external validity of the results. Future studies should increase the sample size to further verify the application effects of the WeChat platform on a larger scale. The WeChat platform relies on smartphones and internet environments, which may prevent some elderly or economically disadvantaged patients from fully utilizing this tool. Therefore, addressing these barriers is one of the key issues that need to be resolved in future research. Although the WeChat platform has shown excellent performance in enhancing patients' self-management abilities and quality of life, its long-term effects still require further observation. Future studies could consider conducting longitudinal follow-up research to evaluate the impact of this management model over extended periods.

**Conclusion**

In summary, the comprehensive management model based on the WeChat platform significantly optimizes the health management of AECOPD patients through personalized health education, real-time symptom monitoring, and convenient doctor-patient interaction. This approach provides new ideas and methods for AECOPD management, demonstrating important clinical application value and promotion potential.

**References**

[1] Kawayama T, Takahashi K, Ikeda T, et al. Exacerbation rates in Japanese patients with obstructive lung disease: A subanalysis of the prospective, observational NOVELTY study. Allergol Int. 2024;73(1):71-80. doi:10.1016/j.alit.2023.08.003
[2] Valle N, Eapen MS, Pillai K, et al. Impact of Nebulized BromAc® on Mucus Plug Clearance in a Mechanically Ventilated Ex Vivo Ovine Lung Model of Obstructive Respiratory Conditions. Life (Basel). 2024;14(9):1111. Published 2024 Sep 3. doi:10.3390/life14091111
[3] Bernardes S, Teixeira PJZ, Silva FM. Association of reduced BMI, length of hospital stay, mortality, and malnutrition diagnosis in patients with acute exacerbation COPD: A secondary analysis of a cohort study. JPEN J Parenter Enteral Nutr. 2023;47(1):101-108. doi:10.1002/jpen.2390
[4] Yang T, Cai B, Cao B, et al. Exacerbation in patients with stable COPD in China: analysis of a prospective, 52-week, nationwide, observational cohort study (REAL). Ther Adv Respir Dis. 2023;17:17534666231167353. doi:10.1177/17534666231167353
[5] Akay B, Bozkurt C, Bulut H. The relationship between mental health continuum and care dependency in individuals with chronic obstructive pulmonary disease: A cross-sectional study. J Adv Nurs. 2024;80(1):301-311. doi:10.1111/jan.15782
[6] Kumari P, Datta P, Gombar S, et al. Epidemiology and clinical outcome of ventilator-associated events at a tertiary care hospital from North India. Trop Doct. 2021;51(2):162-167. doi:10.1177/0049475520982457
[7] Zhou Y, Li C, Chen M, Gao C. Impact of continuous care on quality life of patients with inflammatory bowel disease based on multi-disciplinary cooperation on WeChat platform. Technol Health Care. 2024;32(6):4909-4918. doi:10.3233/THC-232032
[8] Jiang CY, Li CJ, Zhang R, et al. Remote care instruction via the WeChat platform for female patients receiving subcutaneous anticoagulation during the COVID-19 pandemic: a retrospective analysis. PeerJ. 2024;12:e18337. Published 2024 Oct 24. doi:10.7717/peerj.18337
[9] Zhao C, Liang S, Wang X, et al. User experience of a self-management WeChat applet for patients with neurogenic bladder: A qualitative approach. Int J Nurs Sci. 2022;10(1):89-96. Published 2022 Dec 28. doi:10.1016/j.ijnss.2022.12.009
[10] Jin Z, Guo F, Li Y. Effects of Systemic Rehabilitation Nursing Combined with WeChat Publicity and Education on the Early Cognitive Function and Living Quality of the Patients with Cerebral Arterial Thrombosis. J Healthc Eng. 2022;2022:7396950. Published 2022 Feb 24. doi:10.1155/2022/7396950
[11] Wang X, Yan B, Zhang S, et al. Management of volume load for patients undergoing hemodialysis via WeChat and home monitoring in China: a protocol for a cluster-randomized trial. BMC Nephrol. 2025;26(1):58. Published 2025 Feb 6. doi:10.1186/s12882-024-03932-0
[12] McKeon G, Papadopoulos E, Firth J, et al. Social media interventions targeting exercise and diet behaviours in people with noncommunicable diseases (NCDs): A systematic review. Internet Interv. 2022;27:100497. Published 2022 Jan 28. doi:10.1016/j.invent.2022.100497
[13] Ding K, Song F, Qi W, Liu H, Sun M, Xia R. Effects of 12 weeks of head-down strong abdominal breathing on motor and cognitive performance during dual-tasking in patients with chronic obstructive pulmonary disease: Study protocol for a randomised controlled trial. Heliyon. 2024;10(14):e34255. doi:10.1016/j.heliyon.2024.e34255
[14] Jiang Y, Guo J, Chen M, et al. Development of a Pulmonary Rehabilitation Patient Decision Aid for Patients with Chronic Obstructive Pulmonary Disease: Mixed Methods Study. Int J Chron Obstruct Pulmon Dis. 2023;18:1377-1389. doi:10.2147/COPD.S392191
[15] Chinese Medical Association, Chinese Medical Association Publishing House, General Practice Branch of Chinese Medical Association, et al. Chinese Guidelines for Diagnosis and Management of Chronic Obstructive Pulmonary Disease in Primary Care (2024 Edition) Chinese Journal of General Practitioners, 2024, 23(6): 578-602. doi: 10.3760/cma.j.cn114798-20240326-00174.
[16] Saint-Pierre MD. Severe Acute Exacerbations of Chronic Obstructive Pulmonary Disease: Are There Significant Differences Between Hospitalized and Emergency Department Patients?. Int J Chron Obstruct Pulmon Dis. 2024;19:133-138. Published 2024 Jan 16. doi:10.2147/COPD.S447477
[17] Kouraichi C, Sekma A, Bel Haj Ali K, et al. Value of inferior vena cava collapsibility index as marker of heart failure in chronic obstructive pulmonary disease exacerbation. BMC Cardiovasc Disord. 2023;23(1):579. Published 2023 Nov 24. doi:10.1186/s12872-023-03585-1
[18] Bell J, Lim S, Mikami T, et al. The impact on thirty day readmissions for patients hospitalized for acute exacerbations of chronic obstructive pulmonary disease admitted to an observation unit versus an inpatient medical unit: A retrospective observational study. Chron Respir Dis. 2024;21:14799731241242490. doi:10.1177/14799731241242490
[19] Jiang Y, Liu F, Guo J, et al. Evaluating an Intervention Program Using WeChat for Patients With Chronic Obstructive Pulmonary Disease: Randomized Controlled Trial. J Med Internet Res. 2020;22(4):e17089. Published 2020 Apr 21. doi:10.2196/17089
[20] Deng N, Chen J, Liu Y, et al. Using Mobile Health Technology to Deliver a Community-Based Closed-Loop Management System for Chronic Obstructive Pulmonary Disease Patients in Remote Areas of China: Development and Prospective Observational Study. JMIR Mhealth Uhealth. 2020;8(11):e15978. Published 2020 Nov 25. doi:10.2196/15978
[21] Bi J, Yang W, Hao P, et al. WeChat as a Platform for Baduanjin Intervention in Patients With Stable Chronic Obstructive Pulmonary Disease in China: Retrospective Randomized Controlled Trial. JMIR Mhealth Uhealth. 2021;9(2):e23548. Published 2021 Feb 2. doi:10.2196/23548
[22] Lippi L, Turco A, Folli A, et al. Technological advances and digital solutions to improve quality of life in older adults with chronic obstructive pulmonary disease: a systematic review. Aging Clin Exp Res. 2023;35(5):953-968. doi:10.1007/s40520-023-02381-3
[23] Li Y, Qian H, Yu K, et al. The Long-Term Maintenance Effect of Remote Pulmonary Rehabilitation via Social Media in COPD: A Randomized Controlled Trial. Int J Chron Obstruct Pulmon Dis. 2022;17:1131-1142. Published 2022 May 11. doi:10.2147/COPD.S360125
