# Supplementary material for: WeChat-assisted strategies for personalized health management in patients with AECOPD: A randomized controlled trial
Source: PLoS One. 2025 Aug 18;20(8):e0330673. doi: 10.1371/journal.pone.0330673 (PMC12360646; doi:10.1371/journal.pone.0330673)
Supplement: Attachment — Submitted filename: Reviewer Comments.docx [file pone.0330673.s004.docx]

**Reviewer Comments**

This study addresses a relevant and timely topic, leveraging digital platforms (WeChat) for improving personalized health management in patients with Acute Exacerbation of Chronic Obstructive Pulmonary Disease (AECOPD). The manuscript presents a randomized controlled trial with encouraging findings regarding improved self-management, reduced readmissions, and enhanced patient satisfaction. While the study is methodologically sound and provides compelling results, several aspects require clarification or improvement for enhanced rigor and reproducibility.

I. Generally well-written, but a professional language edit is recommended to improve fluency and clarity.
II. Emphasize more clearly in the Introduction how this study goes beyond prior WeChat-based interventions. While the paper cites relevant literature, the novel aspects should be more clearly stated (e.g., longer follow-up, deeper patient-doctor interaction, stratified randomization).
III. The process of blinding (if any) is not mentioned. While blinding may be impractical in behavioral interventions, its absence and possible impact should be discussed. Include CONSORT diagram for transparency in recruitment, allocation, and follow-up (beyond the simple flowchart).
IV. Report effect sizes or risk reductions where appropriate, especially for clinical outcomes like hospital readmission and emergency visits.
V. Figures mentioned (e.g., bar charts for self-management and quality of life scores) are not shown in the provided document. These should be included with proper labeling and statistical annotations. Clarify whether the data were analyzed as intention-to-treat or per-protocol.
VI. Limitations are acknowledged, but the authors could better discuss potential biases (e.g., digital literacy, social desirability in satisfaction reporting) and consider discussing how this model can be adapted for other chronic diseases or in other healthcare settings.

The manuscript is of potential interest to the readership of PLOS ONE. With **minor revisions** addressing the above points, especially in terms of data transparency and methodological reporting, the paper would be suitable for publication.
